# Marine-Derived Metabolites Act as Promising Antifungal Agents

**DOI:** 10.3390/md22040180

**Published:** 2024-04-17

**Authors:** Sijin Hang, Hui Lu, Yuanying Jiang

**Affiliations:** Department of Pharmacy, Shanghai Tenth People’s Hospital, School of Medicine, Tongji University, 200092 Shanghai, China

**Keywords:** marine-derived metabolites, antifungal mechanisms, antifungal targets, invasive fungal diseases

## Abstract

The incidence of invasive fungal diseases (IFDs) is on the rise globally, particularly among immunocompromised patients, leading to significant morbidity and mortality. Current clinical antifungal agents, such as polyenes, azoles, and echinocandins, face increasing resistance from pathogenic fungi. Therefore, there is a pressing need for the development of novel antifungal drugs. Marine-derived secondary metabolites represent valuable resources that are characterized by varied chemical structures and pharmacological activities. While numerous compounds exhibiting promising antifungal activity have been identified, a comprehensive review elucidating their specific underlying mechanisms remains lacking. In this review, we have compiled a summary of antifungal compounds derived from marine organisms, highlighting their diverse mechanisms of action targeting various fungal cellular components, including the cell wall, cell membrane, mitochondria, chromosomes, drug efflux pumps, and several biological processes, including vesicular trafficking and the growth of hyphae and biofilms. This review is helpful for the subsequent development of antifungal drugs due to its summary of the antifungal mechanisms of secondary metabolites from marine organisms.

## 1. Introduction

In contemporary times, the progress made in immune therapy for disease management, encompassing the utilization of immunosuppressive drugs, monoclonal antibody medications, and broad-spectrum antibacterial agents, has resulted in the heightened vulnerability of patients to opportunistic pathogenic fungi [1,2]. Consequently, there has been a rise in the occurrence of lethal invasive fungal diseases (IFDs) among individuals with compromised immune systems [3]. Based on the epidemiological data up to January 2024, it is projected that, annually, more than 6.5 million individuals will be affected by IFDs, resulting in approximately 3.8 million deaths. Of these deaths, approximately 2.5 million can be directly attributed to IFDs. The primary causative species, including *Candida*, *Cryptococcus*, *Pneumocystis*, and *Aspergillus* genera, are responsible for 995,000, 147,000, 214,000, and 1801,000 deaths per year, respectively [4]. Consequently, IFDs are widely recognized as life-threatening infections on a global scale.

Azoles have been widely employed as a therapeutic intervention for the treatment of IFDs [5,6,7]. Furthermore, alternative categories of antifungal agents, including polyenes (such as nystatin (**1**) and amphotericin B (**2**)), flucytosine (**3**), and echinocandins (such as anidulafungin (**4**) and caspofungin (**5**)), have been utilized for the management of IFDs [8,9]. However, drug-resistant fungal pathogens have emerged as a consequence of antifungal treatment. For example, patients undergoing caspofungin (**5**) therapy have exhibited breakthrough infections of *Aspergillus fumigatus* (*A. fumigatus*) and multi-resistant yeasts. In addition, patients receiving voriconazole (**6**) or echinocandin were susceptible to developing mucormycosis and infections caused by other uncommon molds [10]. Therefore, despite the utilization of current antifungal agents, the mortality rates of IFDs remain high, and the prevalence of resistant fungal strains is escalating [11,12,13]. This study found that between 2010 and 2020, 36.8% of *Aspergillus terreus* (*A. terreus*), 14.9% of *Aspergillus flavus* (*A. flavus*), 5.2% of *Aspergillus niger* (*A. niger*), and 2.01% of *A. fumigatus* were identified as amphotericin B-resistant *Aspergillus* species [14]. In addition, there have been reports of azole-resistant *A. fumigatus* strains globally. Specifically, the prevalence of triazole resistance among *A. fumigatus* isolates ranged from 2.9% in Japan to 6.6% in Pakistan between 2011 and 2023 [15]. Therefore, the development of novel antifungal drugs is imperative.

Marine environments have served as a significant platform for the exploration of bioactive compounds. Over 250,000 species, ranging from invertebrates such as sponges and ascidians to microorganisms, have been discovered in these habitats [16]. The unique combination of pressure, salinity, and temperature in the marine environment has resulted in the production of diverse secondary metabolites that differ from those found on land [17]. These marine-derived compounds have exhibited various pharmacological activities, such as antifungal properties [18,19,20]. Furthermore, it has been proven that compounds of marine origin have attractive pharmacokinetic and pharmacodynamic properties that might contribute to treating IFDs caused by drug-resistant fungi [21]. Therefore, marine-derived compounds have great potential for the development of new antifungal drugs. Despite the discovery of numerous compounds with antifungal properties derived from marine sources, the precise mechanisms underlying the antifungal activity of most of these compounds remain elusive [22,23].

This comprehensive review aims to consolidate the knowledge surrounding several marine-derived metabolites that exhibit well-defined antifungal mechanisms. These mechanisms encompass the targeting of various cellular components such as the cell wall, cell membrane, mitochondria, chromosome, drug efflux pumps, and several biological processes, such as vesicular trafficking and the inhibition of hyphal and biofilm growth. This review is beneficial for the subsequent development of antifungal drugs from marine sources.

## 2. Disrupting the Cell Wall

**Figure 1:**
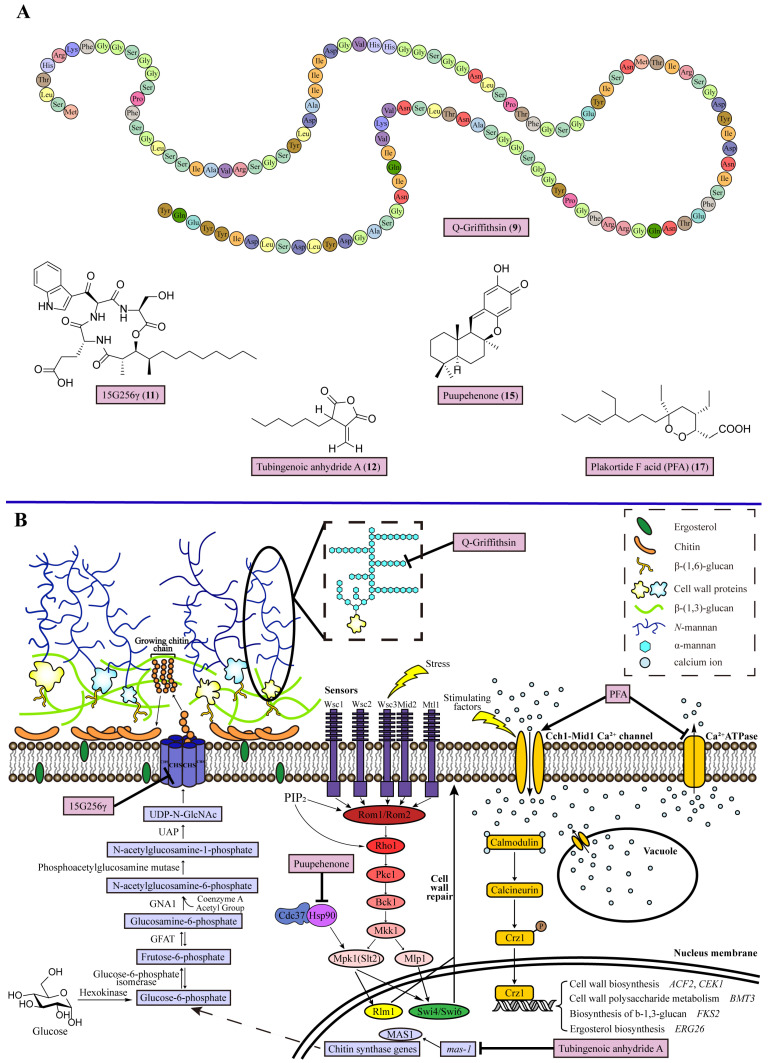
Chemical structures and detailed mechanisms of marine-derived metabolites targeting fungal cell wall. (**A**) Chemical structures of Q-Griffithsin (**9**), 15G256γ (**11**), tubingenoic anhydride A (**12**), puupehenone (**15**), and PFA (**17**). (**B**) Several molecules or processes related to cell wall construction could be attacked by these antifungal metabolites, including mannans in the cell wall, CHS and related genes, CWI pathway, and Ca^2+^ homeostasis. Q-Griffithsin binds to α-mannan, breaking the fibrillar network constructed by *N*-mannans; 15G256γ acts as a CHS inhibitor to block chitin synthesis; tubingenoic anhydride A inhibits *mas-1* gene producing Mas1, which mediates expression of CHS, leading to blocked chitin synthesis; puupehenone occupies a binding site on Hsp90 involved in Hsp90–Cdc37 interaction to disrupt Hsp90 activity for Slt2 activation in CWI pathway; PFA involves disruption of Ca^2+^ homeostasis in fungi through inhibiting the activity of Ca^2+^ ATPase or activating Ca^2+^ channels, which results in over-accumulated Ca^2+^ in intracellular environment.

The fungal cell wall serves as a means of mechanical protection for fungi, ensuring the maintenance of osmotic pressure and cellular shape. Moreover, it plays a crucial role in fungal pathogenicity and virulence, facilitating host invasion during IFDs [24]. In addition, the cell wall distinguishes fungal cells from those of humans and other mammals, making it a more selective and less toxic target for antifungal strategies [25]. The fungal cell wall could be divided into two distinct layers, namely, the inner wall and the outer wall. Taking *Candida albicans* (*C. albicans*) as an example, the inner cell wall is composed of chitin interspersed with β-(1,3)-glucan. Within the inner layer, mannosylated cell wall proteins (CWPs) are distributed and covalently linked to chitin and β-(1,3)-glucan via β-(1,6)-glucan linkages. The outer layer of the cell wall is formed by attaching *N*-mannan structures to CWPs, while short-chain *O*-mannan are also attached to CWPs [26] (Figure 1).

### 2.1. Inhibiting Mannan Biosynthesis

*N*-mannan possesses distinct structural components, including an inner core, a backbone composed of α-mannan, α-mannan side chains, and outer β-oligomannosides. The inner core is composed of two *N*-acetylglucosamine groups, while the backbone consists of α-mannan linked through 1,6 bonds. The addition of β-oligomannosides to the backbone occurs via α-1,2-glycosidic bonds. The length of the mannose α-1,2-linked side chain can be increased by incorporating one or more α-1,3-linked mannose units. Therefore, α-mannan plays a crucial role as a fundamental constituent of *N*-mannan [27].

Mannan plays a significant role in the virulence of fungi. Within *C. albicans*, the genes *MNT1* and *MNT2* partially encode α-1,2-mannosyltransferases, which are responsible for the second and third mannose residues in *O*-mannan [28]. When the *MNT1* and *MNT2* genes are deleted in *C. albicans*, either individually or in combination, the *O*-mannan structure is truncated. The absence of the *MNT1* and *MNT2* genes leads to a diminished ability to form the cell wall and adhere to host surfaces in *C. albicans*, resulting in attenuated virulence [28]. The genes *MNS1*, *CWH41*, and *ROT2* are responsible for encoding three enzymes found in the endoplasmic reticulum, namely, α-glucosidase I, the α-glucosidase II catalytic submit, and α-1,2-mannosidase. These enzymes play a crucial role in *N*-glycan core processing for the biosynthesis of *N*-mannan that takes place within the endoplasmic reticulum. When these genes are knocked out in *C. albicans*, it results in a reduction in the *N*-mannan content and an increase in flocculation, thereby disrupting the cell wall integrity and host–fungus interactions, leading to virulence loss [29]. Pradimicin A (**7**) exhibits strong antifungal activity against *C. albicans*, *A. fumigatus*, and *Cryptococcus neoformans* (*C. neoformans*), with minimum inhibitory concentration (MIC) values ranging from 1.6 to 12.5 µg/mL. This activity is attributed to its calcium ion (Ca^2+^)-dependent binding to fungal cell-surface mannan, leading to the generation of reactive oxygen species (ROS) and inducing apoptosis-like cell death. Pradimicin A (**7**) has been utilized for the prophylaxis and treatment of opportunistic fungal infections in individuals with acquired immune deficiency syndrome (AIDS) [30,31].

Griffithin (**8**), derived from red alga *Griffithsia* species, exhibits wide-ranging antiviral properties against human immunodeficiency virus and other viruses [32]. Nonetheless, the surface Met78 residue in Griffithin (**8**) is vulnerable to oxidation. To enhance the stability of Griffithin (**8**), the substitution of the Met78 residue with glutamine (Q) was performed, resulting in the creation of a recombinant lectin known as Q-Griffithsin (**9**) (Figure 1). Remarkably, the previously unknown antifungal activity of Q-Griffithsin (**9**) has been uncovered. The MIC values for Q-Griffithsin (**9**) against various *Candida* species, including *C. albicans*, *Candida glabrata* (*C. glabrata*), *Candida parapsilosis* (*C. parapsilosis*), *Candida krusei* (*C. krusei*), and *Candida auris* (*C. auris*), were determined to be 6, 95, 24, 95, and 48 mg/mL, respectively. Q-Griffithsin (**9**) also has significant preventive and therapeutic activity in murine models of vaginal candidiasis [33]. Notably, the potent MIC value observed for Q-Griffithsin (**9**) against *C. albicans* can be attributed to its exceptional binding capability [32]. Further studies have indicated that Q-Griffithsin (**9**) binds to α-mannan on the cell wall of *Candida* species with a 50% effective concentration (EC_50_) of 23.47 ng/mL. The binding between Q-Griffithsin (**9**) and α-mannan breaks the outer layer of the fungal cell wall, which has a dense fibrillar network constructed by N-mannans, resulting in increased wall porosity and permeability (Figure 1). Therefore, Q-Griffithsin (**9**) disrupts cell wall integrity, causing desiccation and loss of fungal budding ability (Table 1).

### 2.2. Inhibiting Chitin Biosynthesis

Chitin is a β-1,4-linked linear polysaccharide with rigidity and non-elasticity, and is regarded as one of the strongest biomaterials in nature. In the inner wall, rigid chitins intersperse with flexible β-(1,3)-glucans to exhibit enough strength to resist outwardly directed turgor pressure [8,26]. Inhibiting chitin biosynthesis is a promising antifungal strategy, as chitin is absent in humans [34]. The process of chitin biosynthesis involves the conversion of glucose or glycogen into the primary constituent of fungal chitin, diphosphate-N-acetylglucosamine (UDP-*N*-GlcNAc), within the intracellular space. Subsequently, linear chitin chains are secreted extracellularly to form fungal cell walls. Initially, glucose is transformed into glucose-6-phosphate through hexokinase. Glucose-6-phosphate is then isomerized into fructose-6-phosphate by isomerase and then converted into glucosamine-6-phosphate by glutamine fructose-6-phosphate amidotransferase (GFAT). An acetyl group is added through glucosamine-6-phosphate acetyltransferase (GNA1) to generate *N*-acetylglucosamine-6-phosphate, which is further converted into *N*-acetylglucosamine-1-phosphate by the catalytic action of phosphoacetylglucosamine mutase (AGM-1). Finally, UDP-*N*-acetylglucosamine pyrophosphorilase (UAP) catalyzes the formation of UDP-*N*-GlcNAc. UDP-*N*-GlcNAc, as a substrate of chitin synthase (CHS), is further catalyzed by CHS to transform GlcNAc from UDP-*N*-GlcNAc into an emerging chitin chain and directs the novel chitin chain to the extracellular space for incorporation into the cell wall (Figure 1).

Chitin synthase (CHS) is regarded as the key enzyme in chitin biosynthesis, and could be the target for inhibiting chitin biosynthesis in fungi [35]. The genes of the CHS family can be grouped into seven classes (I to VII). The seven classes of CHSs possess varying domain structures. CHSs from class VI have the simplest protein domain structure, Chitin_synth_2 (CS 2 domain), which is the conserved core domain of the CHSs. The CHSs in classes I, II, and III replace the CS 2 domain with the CS 1 domain. For classes IV, V, and VII, these CHSs share the gain of a cytb5-like binding domain [36,37]. CHSs with various classes play different roles in fungal cells, and most fungi have multiple genes that encode CHSs [38,39]—for example, the members of *CHS* in *Saccharomyces cerevisiae* (*S. cerevisiae*) include *CHS* I, *CHS* II, and *CHS* IV. *CHS* I acts as a repair enzyme to replenish chitin in the cell wall after cell division; *CHS* II participates in the processes of primary septum formation and cell division, while *CHS* IV is responsible for the majority of chitin synthesis. *C. albicans* has four *CHS* copies: two copies of *CHS* I, one copy of *CHS* II, and another of *CHS* IV [40]. Nikkomycin Z (**10**) has been identified as a selective competitive inhibitor of CHS III in *S. cerevisiae*, functioning as a competitive analogue of the CHS substrate UDP-N-GlcNAc [41]. Studies have indicated that the MIC values of nikkomycin Z (**10**) are 4 and 2 μg/mL for *C. albicans* and *C. parapsilosis*, respectively [42]. Furthermore, results from a human Phase 1 trial suggest that nikkomycin Z (**10**) does not raise safety concerns [43].

A cyclic lipopeptide antifungal compound 15G256γ (**11**), produced by the marine fungus *Hypoxylon oceanicum*, was discovered in a screen for antifungal agents with cell wall-impacting mechanisms [44]. 15G256γ (**11**) exhibits broad-spectrum activity against human fungal pathogens with values of the MIC ranging from 2 to 16 μg/mL for dermatophytic fungi, including *Trichophyton rubrum* (*T. rubrum*), *Trichophyton mentagrophytes* (*T. mentagrophytes*), *Epidermophyton floccosum* (*E. floccosum*), *Microsporum audoinii* (*M. audoinii*), *C. albicans*, *C. parapsiliosis*, and *C. glabrata*. Under a high concentration of 15G256γ (**11**) (~400 μg/mL), the activity of the in vitro *Neurospora crassa* CHS decreased clearly, proving the CHS-inhibiting activity of 15G256γ (**11**). Then, after *Cochliobolus sativus* (*C. sativus*) fungus was treated with 250 μg/mL of 15G256γ (**11**) for 48 h, the hyphae had a ‘beaded’ appearance and produced many highly swollen protoplast-like structures, suggesting the loss of chitin possibly weakened the cell walls, causing hyphal bulging (Table 1).

Tubingenoic anhydride A (**12**), the metabolite of the fungus *Aspergillus tubingensis* (*A. tubingensis*) OY907 from the sponge *Ircinia variabilis*, shows inhibitory activity against *Neurospora crassa* (*N. crassa*) growth, with an MIC value of 330 μM [45]. Using a genetic approach, the results revealed that tubingenoic anhydride A (**12**) inhibits the expression of the gene Marine-Aspergillus Sensitive 1 (*mas-1*), whose product mediates at least part of the expression of CHS. The *A. tubingensis* strain with a Δ*mas-1* deletion showed two-fold more resistance to the CHS inhibitor Polyoxin D (**13**) than the wild type. Moreover, the expression of the CHS genes in the Δ*mas-1 N. crassa* strain is highly elevated, and is two-fold higher than that in the wild type in all the *CHSs*. The *CHSs* in *N. crassa* are composed of *CHS* I, *CHS* II, *CHS* III, *CHS* IV, *CHS* V, *CHS* VI, and *CHS* VII. Of them, *CHS* IV has the highest expression levels compared with the other *CHS*s, and its product is regarded as an auxiliary enzyme under stressful conditions in *N. crassa*. So, tubingenoic anhydride A (**12**) interferes with *mas-1* expression, leading to an effect on the constitutive expression of *CHS* to disrupt cell wall integrity (Table 1).

### 2.3. Inhibiting the Cell Wall Integrity (CWI) Pathway

The CWI pathway regulates the repair of the cell wall in response to various environmental stresses. In *S. cerevisiae*, the initiation of the CWI pathway is facilitated by cell-surface sensors, including Wsc1, Wsc2, Wsc3, Mid2, and Mtl1, which detect cell wall stress. These sensors, along with phosphatidylinositol 4,5-bisphosphate (PIP_2_), work together to recruit the guanosine nucleotide exchange factors (GEFs) Rom1 and Rom2 to the plasma membrane. Subsequently, three GEFs, Rom1, Rom2, and Tus1, in conjunction with additional PIP_2,_ stimulate nucleotide exchange in Rho1. Rho1 initiates the activation of protein kinase C (Pkc1) to lead a cascade of mitogen-activated protein kinase (MAPK) signaling involving Bck1, Mkk1, Mpk1 (Slt2), and Mlp1 activation in sequential order. Subsequently, Mpk1 (Slt2) and Mlp1 contribute to a transcriptional program. Within the nucleus, three transcription factors, namely, Rlm1, Swi4, and Swi6, become activated and assume responsibility for regulating the expression of genes involved in cell wall biosynthesis [46]. The activation of protein kinases involved in MAPK signaling relies on their association with other proteins, including Hsp90 and Cdc37 [47]. Hsp90 is a conserved eukaryotic molecular chaperone that plays a key role in the CWI signaling pathway and contributes to cell wall integrity [48]. During temperature stress, Hsp90 stabilizes the key proteins Pkc1 and Mpk1 (Slt2) in the MAPK signaling cascade, reinforcing the function of the CWI pathway, which is based on Cdc37, the main cochaperone of Hsp90, targeting client proteins such as Mpk1 (Slt2) to the chaperone Hsp90, interacting physically with Hsp90, and regulating the ATPase activity of Hsp90 [48,49]. The phosphorylation of Ser14 on Cdc37 is important for efficient interaction with Hsp90, which is a highly conserved portion of the client-binding domain of Cdc37 [49]. Therefore, Hsp90 and Cdc37 act as the regulators of the CWI pathway, which is necessary for Slt2 activation and downstream transcription factor Rlm1 (Figure 1). Clotrimazole (**14**), a synthetic imidazole derivative known for its broad-spectrum antifungal properties, has been identified by Sellers-Moya et al. as potentially inducing alterations in MAPK signaling, leading to the reconfiguration of the CWI signaling pathway in *S. cerevisiae* [50].

Puupehenone (**15**), a sesquiterpene quinone derived from marine sponges, has been found to significantly enhance the effectiveness of caspofungin against fungal pathogens [51]. When combined, puupehenone (**15**) and caspofungin (**5**) exhibit a synergistic effect against caspofungin-insensitive *C. neoformans*, as well as caspofungin-resistant strains of *C. glabrata* and *C. albicans*, with a fractional inhibitory concentration index (FICI) value of 0.38, 0.48, and 0.39, respectively [51]. The mechanistic characterization of puupehenone (**15**) in *S. cerevisiae* demonstrated that puupehenone (**15**) prevented cell wall repair through the CWI signaling pathway and interfered with the activity of Hsp90 [51]. Molecular modeling has predicted that puupehenone (**15**) occupies a binding site on Hsp90 that is involved in the interaction between Hsp90 and Cdc37, which potentially impairs CWI pathway signaling. The likely target site for puupehenone (**15**) is Gln-119 in the polar pocket of Hsp90 surrounded by Gln-119, Arg-32, and Asn-37. Since the Hsp90–Cdc37 interaction is blocked, the client protein kinase Mpk1 (Slt2) cannot be activated and then, in turn, the induction of Rlm1 for fungal cell wall repair and maintenance is inhibited. In conclusion, puupehenone (**15**) acts as an Hsp90 inhibitor, which prevents Hsp90–Cdc37 interaction, resulting in the deactivation of Mpk1 (Slt2) and downstream Rlm1 to cut off the CWI pathway [51] (Table 1).

### 2.4. Disrupting Ca^2+^ Homeostasis

In fungal cells, Ca^2+^ homeostasis should be strictly controlled, which plays an essential role in cell wall biosynthesis [52,53]. This homeostasis system is composed of various calcium channels, calcium pumps (Ca^2+^ ATPases), and calcineurins, of which calcineurins are the primary regulators of Ca^2+^ homeostasis in fungal cells. Taking *C. albicans,* for example, stimulating factors activate the Cch1-Mid1 Ca^2+^ channel on the plasma membrane, leading to the influx of extracellular Ca^2+^. Meanwhile, the internally stored Ca^2+^ is released from vacuoles into the cytoplasm. The increased intracellular Ca^2+^ levels in the cytoplasm are received by calmodulin, causing the activation of calcineurin. Then, the downstream target Crz1, a transcription factor regulating the genes of cell wall biosynthesis, is dephosphorylated by activated calcineurin, promoting its expression [5]. Moreover, in terms of Crz1, besides regulating the genes for cell wall biosynthesis, such as *ACF2* and *CEK1*, there are also studies reporting its regulation of genes for cell wall polysaccharide metabolic processes (e.g., *BMT3*), the biosynthesis of β-1,3-glucan (*FKS2*), and the process of ergosterol biosynthesis (*ERG26*) [54]. Hence, the manipulation of Ca^2+^ homeostasis presents a potential alternative approach for impeding cell wall biosynthesis as an antifungal strategy (Figure 1). The clinical antiarrhythmic drug amiodarone (**16**) has demonstrated fungicidal properties against various pathogenic fungi, such as species of *Cryptococcus*, *Saccharomyces*, *Aspergillus*, *Candida*, and *Fusarium* [55]. The antifungal mechanism of amiodarone involves the disruption of Ca^2+^ homeostasis, leading to a rapid and substantial influx of Ca^2+^ and subsequent cytoplasmic Ca^2+^ overload [56].

Plakortide F acid (PFA) (**17**) is a polyketide endoperoxide derived from the marine sponge *Plakortis halichondrioides* with a significant inhibitory effect against *C. albicans*, *C. neoformans*, and *A. fumigatus* with MIC values of 0.08, 2.5, and 5.00 μg/mL, respectively [57]. PFA-treated fungal cells show a significant rise in intracellular Ca^2+^ levels. Meanwhile, fungal mutants lacking Ca^2+^ transporters or calcineurin function exhibit increased antifungal activity. For the other mode of action of PFA (**17**) seen in *S. cerevisiae*, there could be two possible explanations. One is that PFA (**17**) inhibits the activity of Ca^2+^ ATPases to inhibit Ca^2+^ efflux, leading to excess Ca^2+^ accumulation in the cytosol, inducing apoptosis. The other might be due to the influx of Ca^2+^ caused by its interaction with the plasma membrane [58]. When PFA (**17**) intercalates into the fungal membrane, the presence of the long hydrocarbon chain and carboxylic group in PFA (**17**) alters the lipid fluidity. It induces the hyperpolarization of the plasma membrane, activating hyperpolarization-activated Ca^2+^ channels, causing Ca^2+^ influx into the cytosol, disrupting Ca^2+^ homeostasis, and leading to cell death (Figure 1). Taken together, PFA (**17**) can be said to target Ca^2+^ homeostasis for antifungal activity (Table 1).

## 3. Disrupting the Cell Membrane

The cell membrane of fungi serves a crucial function in determining cell shape and facilitating various cellular processes, including the stress response, cell recognition, signal transduction, apoptosis, and pathogenicity [59]. A rich composition of diverse lipids and membrane proteins characterizes fungal cell membranes. Among the lipids present, glycerophospholipids, sphingolipids, and sterols, particularly ergosterol, are prominent in fungal cells. Given the importance of fungal cell membranes, disrupting cell membranes is a feasible antifungal strategy [60].

### 3.1. Targeting Ergosterol in the Cell Membrane

Ergosterol, with the structure of a 3β-OH group, is an effective target for treating IFDs. For example, amphotericin B (**2**) is a broad-spectrum antifungal agent in clinical settings. This kind of antifungal agent binds with ergosterol, forming a drug–ergosterol complex, which is incorporated into fungal cell membrane-forming channels, causing osmotic cell lysis and disrupting physiological ion transport [61]. Theonellamides are distinctive antifungal bicyclic dodecapeptides from *Theonella* species of marine sponges, which represent a novel class of sterol-binding compounds whose mode of action differs from amphotericin B (**2**) [62]. Theonellamide G (**18**), a member of the theonellamide family, exhibits potent antifungal activity towards amphotericin B-resistant strains of *C. albicans,* with an IC_50_ of 2.0 μM [63]. Further studies of its mechanism have shown the kinetics of Theonellamide G binding to the membrane are consistent with the two-state reaction model, as it binds to the membrane surface and forms a stable membrane complex. Theonellamide G (**18**) preferentially binds to 3β-OH-containing membranes, and further studies have proven there is a direct interaction between Theonellamide G (**18**) and 3β-OH groups. Unlike amphotericin B (**2**), Theonellamide G (**18**) does not form distinct pores on the plasma membrane but disturbs and damages the membrane morphology and integrity through its accumulation. This is because theonellamide mostly recognizes the 3β-OH group of the sterol in the first step and the second step of Theonellamide G’s (**18**) action has a weaker dependency on the sterol [62] (Table 1).

**Figure 2:**
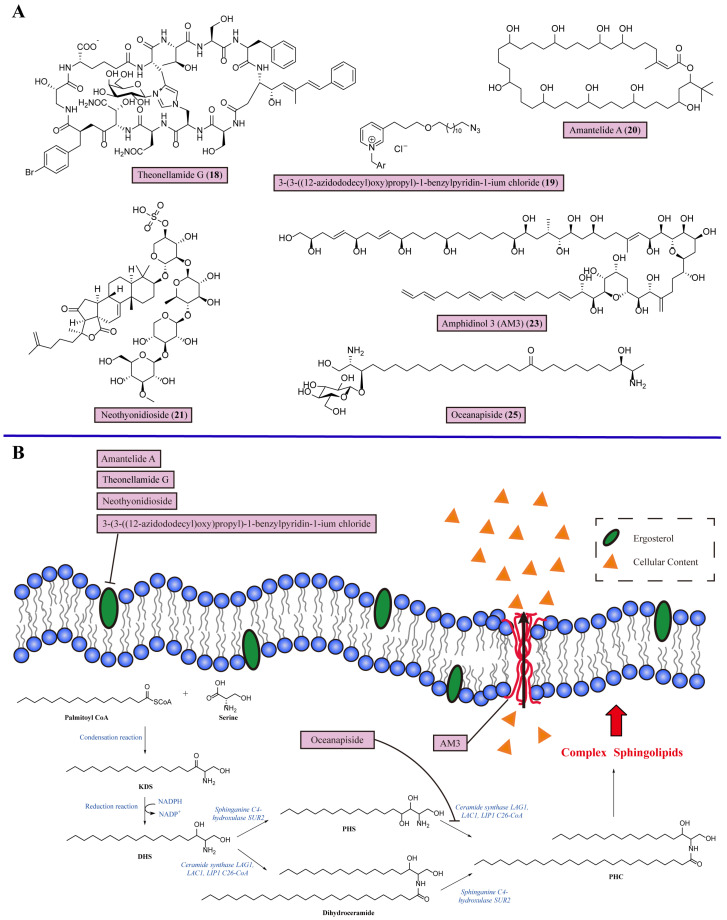
Chemical structures and detailed mechanisms of marine-derived metabolites targeting fungal cell membrane. (**A**) Chemical structures of Theonellamide G (**18**), 3-(3-((12-azidododecyl)oxy)propyl)-1-benzylpyridin-1-ium chloride (**19**), amantelide A (**20**), neothyonidioside (**21**), AM3 (**23**), and oceanapiside (**25**). (**B**) The mechanisms involved in these active metabolites are as follows: targeting of ergosterol on the cell membrane, membrane permeabilization, and sphingolipid biosynthesis. Theonellamide G directly binds with the 3β-OH group in ergosterol to cause accumulation of Theonellamide G on the membrane, causing membrane damage; 3-(3-((12-azido-dodecyl)oxy)propyl)-1-benzylpyridin-1-ium chloride interacts with ergosterol to perform its antifungal function; amantelide A recognizes ergosterol without depending on its 3β-OH group to disrupt the fungal membrane; neothyonidioside forms a large complex by binding with ergosterol to suppress the membrane’s ability to bend and produce multivesicular body vesicles in fungal cells; AM3 locates the fungal membrane through the 3β-OH group of ergosterol and forms a pore in the membrane through its hairpin configuration; oceanapiside inhibits sphingolipid biosynthesis at the step of the conversion of PHC to PHS by the competitive inhibition of ceramide synthase Lag1, Lac1, or Lip1.

The marine pyridine alkaloid derivative 3-(3-((12-azidododecyl)oxy)propyl)-1-benzylpyridin-1-ium chloride (**19**) is distributed widely in marine sponges of the Haplosclerida order [64]. This alkaloid (**19**) inhibits the growth of *Candida* species, and its rapid fungicidal effect against *C. albicans* ATCC10231 was evaluated in vitro at 7.8 and 15.6 μg/mL. The *C. albicans* cells treated with this alkaloid (**19**) displayed obvious invaginations on their fungal membranes. Further, the exogenous ergosterol experiment proved that it binds to the membrane ergosterol of *C. albicans* to perform its antifungal function (Figure 2, Table 1).

Amantelide A (**20**), the polyhydroxylated macrolactone isolated from marine gray cyanobacteria Oscilliatoriales, displays different interactions with fungal membranes compared with other antifungals [65]. Amantelide A (**20**) inhibits the growth of *S. cerevisiae* and *Schizosaccharomyces pombe* (*S. pombe*) with MIC values of 50 and 12.5 μM, respectively. In a culture of *S. cerevisiae* with an ergosterol biosynthesis (*EGR*) mutant, amantelide A (**20**) maintained its antifungal sensitivity against *erg6*Δ *S. cerevisiae*, indicating that the antifungal activity of amantelide A (**20**) does not completely depend on binding with ergosterol. The results showed that amantelide A (**20**) significantly binds with model membranes based on artificial liposomes. Moreover, the increased binding of amantelide A (**20**) was observed with membranes containing ergosterol. Therefore, amantelide A (**20**) enables the recognition of sterol molecules on the plasma membrane, and the presence of ergosterol could increase its membrane binding activity (Figure 2). Compared with other sterol-targeting antifungals like nystatin, amantelide A (**20**) functions independent of the 3β-OH group, the partial structure of ergosterol utilized for lipid recognition. Amantelide A (**20**) has the structural flexibility to recognize various sterol molecules. Alternatively, the sterol molecules affect the stability of amantelide A (**20**) by changing the surrounding conditions of the fungal membrane (Table 1).

Neothyonidioside (**21**), a triterpene glycoside, is from the sea cucumber *Australostichopus mollis* [66]. It has comparable potency to polyene antifungals, with an MIC of 1 μM against *S. cerevisiae*. Ergosterol is involved in neothynidioside’s (**21**) mode of action. Adding exogenous ergosterol to *S. cerevisiae* reversed the antifungal activity of neothynidioside (**21**), suggesting that neothynidioside (**21**) binds directly to ergosterol. Then, an inhibitor of *ERG11*, ketoconazole, which can reduce membrane ergosterol levels, was used to perform a drug interaction experiment. Ketoconazole (**22**) induced the resistance of *S. cerevisiae* against neothynidioside (**21**) in a dose-dependent manner, showing that the activity of neothynidioside (**21**) requires a certain minimum threshold level of ergosterol. This result proves that neothynidioside (**21**) forms a large complex with ergosterol, exhibiting a cooperative effect. Interestingly, neothynidioside (**21**) binds to ergosterol and does not permeabilize the cell membrane but does act on endocytosis depending on the sterol (Figure 2). The sterol-like core of neothynidioside (**21**) imparts rigidity to the plasma membrane when binding to ergosterol, reducing the ability to bend and form multivesicular body vesicles for antifungal activity [66] (Table 1).

Amphidinols (AMs) are a family of unique dinoflagellates with strong antifungal abilities, which have been isolated from the dinoflagellate *Amphidinium klebsii*. Their structures are best characterized as a long carbon chain encompassing multiple hydroxyl groups and polyolefins, which take a hairpin configuration in the membrane. AMs have potent membrane permeabilizing abilities, and their intricate molecular mechanism depends on the polyolefins to bind the bilayer as well as the multiple hydroxyl groups formed by the pore in the membrane [67]. Among the AM homologs, amphidinol 3 (AM3) (**23**) has shown a significantly greater antifungal effect, with a minimal effective concentration (MEC) of 9.0 μg/disk against *A*. *niger* [68]. Light scattering experiments have shown that the activity of AM3 (**23**) is mainly due to the polyene moiety of AM3 (**23**) binding to the lipid core, leading to fungal membrane permeabilization (Figure 2). Further studies have revealed that the membrane permeabilizing activities of AM3 (**23**) depend on its interaction with sterols and the strict stereospecific recognition of the structure of the 3-OH group of ergosterol, probably through hydrogen bonding, which enhances the membrane-binding efficiency of AM3 (**23**) to permeabilize the fungi plasma membrane without altering the membrane integrity. It is supposed that the middle region of AM3 (**23**) containing two tetrahydropyran rings might be responsible for recognizing the 3β-OH group in the sterol [69] (Table 1).

### 3.2. Inhibiting Sphingolipid Biosynthesis

As the sphingolipid is one of the basic components of the plasma membrane, sphingolipid biosynthesis is necessary for the formation of cellular membranes. The steps of sphingolipid biosynthesis in fungi are described as follows. The process starts in the endoplasmic reticulum. First, the condensation of palmitoyl-coenzyme A (CoA) and serine synthesizes 3-ketodihydrosphingosine (KDS) following the nicotinamide adenine dinucleotide phosphate (NADPH)-dependent reduction into dihydrosphingosine (DHS). Then, DHS undergoes C4 hydroxylation through the action of Sur2 to form phytosphingosine (PHS) and is changed into phytoceramide (PHC) by the action of ceramide synthases. In addition, DHS could also be catalyzed by ceramide synthases first, then undergo C4 hydroxylation to synthesize PHC. Furthermore, PHC is transported to the Golgi for the formation of complex sphingolipids. The IPC synthase AUR1 catalyzes the synthesis of inositol-phosphoramide (IPC) via the addition of phosphatidylinositol. By adding mannose from guanosine diphosphate (GDP)-mannose to IPC through mannose-inositol phosphoceramide (MIPC) synthase, MIPC is synthesized. Mannose (inositol-P)_2_ceramide (M(IP)_2_C) is synthesized with the addition of a second phosphatidylinositol to MIPC, catalyzed by inositol phosphotransferase. Finally, these complex sphingolipids, IPC, MIPC and M(IP)_2_C, are transported to the plasma membrane [70,71] (Figure 2). Due to the structural differences in sphingolipid synthesis between fungi and humans, the biosynthesis of fungal sphingolipids has become a promising target for the development of novel antifungal agents. *N*′-(3-bromo-4-hydroxybenzylidene)-2-methylbenzohydrazide (**24**) has demonstrated the selective inhibition of fungal glycosphingolipids, exhibiting significant in vitro activity against *Cryptococcus* species, including fluconazole-resistant strains [72]. With promising results, this compound is poised for clinical trials [73].

Oceanapiside (**25**) is isolated from the marine sponge *Oceanapia phillipensis* and has a novel chemical structure: a glycosylated “two-headed” α, ω-*bis*-amino alcohol binding structure with similar functional head groups of sphinganine at the terminal (Figure 2). Oceanapiside (**25**) exhibits potent selective activity against *C. glabrata* with an MIC of 10 µg/mL [74]. Biochemical analyses have demonstrated that oceanapiside (**25**) targets the sphingolipid pathway in pathogenic *C. glabrata*. Oceanapiside (**25**) treatment with exogenous 3 µg/mL PHS eliminated antifungal activity against *C. glabrata,* but not with exogenous KDS or DHS, indicating that oceanapiside (**25**) targets the downstream step in sphingolipid biosynthesis and does not affect the initial steps of the assembly of the intermediates KDS and DHS. Then, liquid chromatography–electrospray ionization-mass spectrometry was used to measure the quantity of KDS, DHS, PHS, and PHC in *C. glabrata* with 10 µg/mL of oceanapiside, in which the PHS level showed a remarkable increase (6.5-fold) and the PHC level remained constant compared with the untreated group. This result showed that the conversion of PHS to PHC is blocked, thereby resulting in the accumulation of PHS. The remaining PHC suggested that the synthesis of PHC from DHC was unaffected. Therefore, a reasonable explanation for the oceanapiside (**25**) antifungal mechanism is the competition between oceanapiside (**25**) and PHS as substrates for the ceramide synthase Lag1, Lac1, or Lip1 to block sphingolipid biosynthesis in *C. glabrata* [75] (Figure 2, Table 1).

## 4. Effects on Fungal Chromosomes

The effect of metabolites on DNA functions could result in a series of disturbances in intracellular events, blocking the replication and transcription of DNA, thereby leading to the termination of protein processing for necessary biological processes in cells, such as respiratory metabolism, membrane biosynthesis, and intercellular communication, eventually leading to the death of fungal cells [76]. Flucytosine (**3**), a pyrimidine antifungal approved in the 1960s, has the potential to be metabolized into the toxic compound 5-fluorouracil by fungal cytosine deaminase, thereby disrupting RNA and DNA metabolism. Despite its efficacy, the emergence of drug resistance with flucytosine (**3**) treatment has restricted its utility in clinical settings [77].

**Figure 3:**
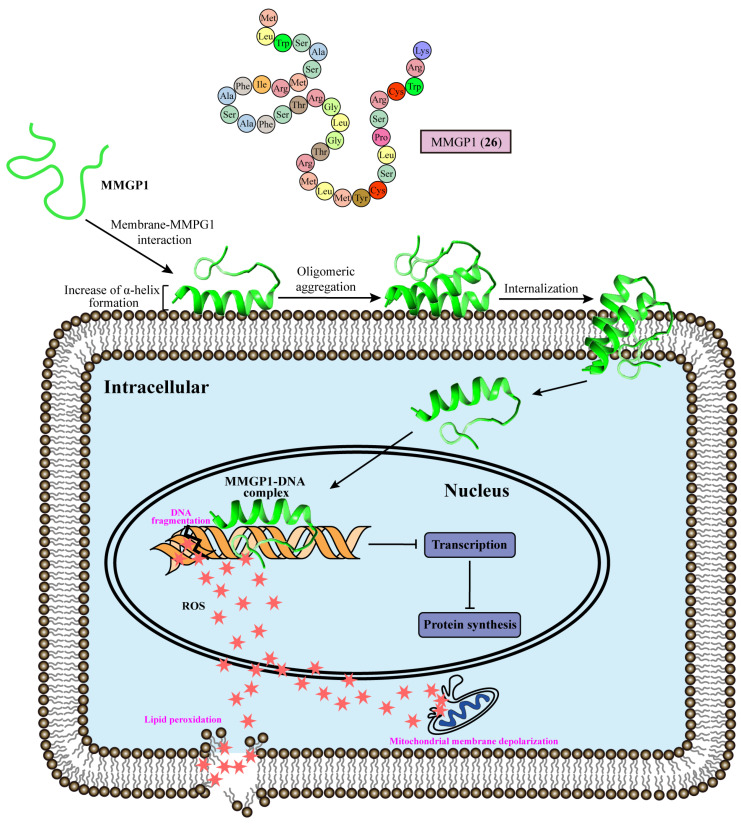
Amino acid sequence and antifungal action of MMGP1 (**26**). MMGP1 first adsorbs to the membrane and forms an α-helical structure. Upon undergoing conformational dynamics, MMGP1 forms an oligomeric aggregate to penetrate the cell membrane and enter the nucleus, where MMGP1 binds with DNA to stop cellular transcription. This leads to hyperproduction of ROS, triggering cascade events of DNA fragmentation, mitochondrial membrane depolarization, and lipid peroxidation, resulting in cell death.

The antifungal peptide MMGP1 (**26**) consists of 36 amino acids from the marine metagenome with MIC values of 0.57 μM against *C. albicans*. MMGP1 (**26**) could be internalized into the cytosol via a time-dependent and energy-independent mechanism, acting as a cell-penetrating peptide [78]. There are three putative hot spot regions of residues 1–5, 7–15, and 21–29 in MMGP1 (**26**), which can nucleate the aggregation process on the cell membrane to form oligomeric aggregates. In a membrane–MMPG1 interaction study, fungal cell membrane lipid bilayers containing a greater proportion of anionic lipids (>60%) induced an increase in the α-helical content formation of MMGP1 (**26**), facilitating the insertion of MMGP1 (**26**) into the plasma membrane [79]. Further studies have revealed the intracellular functions of of MMGP1 (**26**) in *C. albicans* [80]. MMGP1 (**26**) forms peptide–DNA complexes inside the fungal cell, which interfere with the transcription process and macromolecular synthesis. Docking analyses have studied the interaction of MMGP1 (**26**) and B-DNA, the most common DNA molecule in vivo. MMGP1 (**26**) interacts with DNA through hydrophobic and hydrophilic interactions via the following DNA-binding amino acid residues: TRP3, SER4, MET7, ARG8, PHE10, ALA11, GLY20, THR21, ARG22, MET23, TRP34, and LYS36 [81]. Moreover, MMGP1 binds to DNA, which inhibits the transcription process within *C. albicans*, thereby inducing the endogenous production of ROS, causing lipid peroxidation, mitochondrial membrane depolarization, and DNA fragmentation (Figure 3). In conclusion, the marine peptide MMGP1 (**26**) directly interacts with DNA in *C. albicans*, triggering a cascade of events that lead to cytotoxicity against *C. albicans* (Table 1).

## 5. Mitochondrial Dysfunction

**Figure 4:**
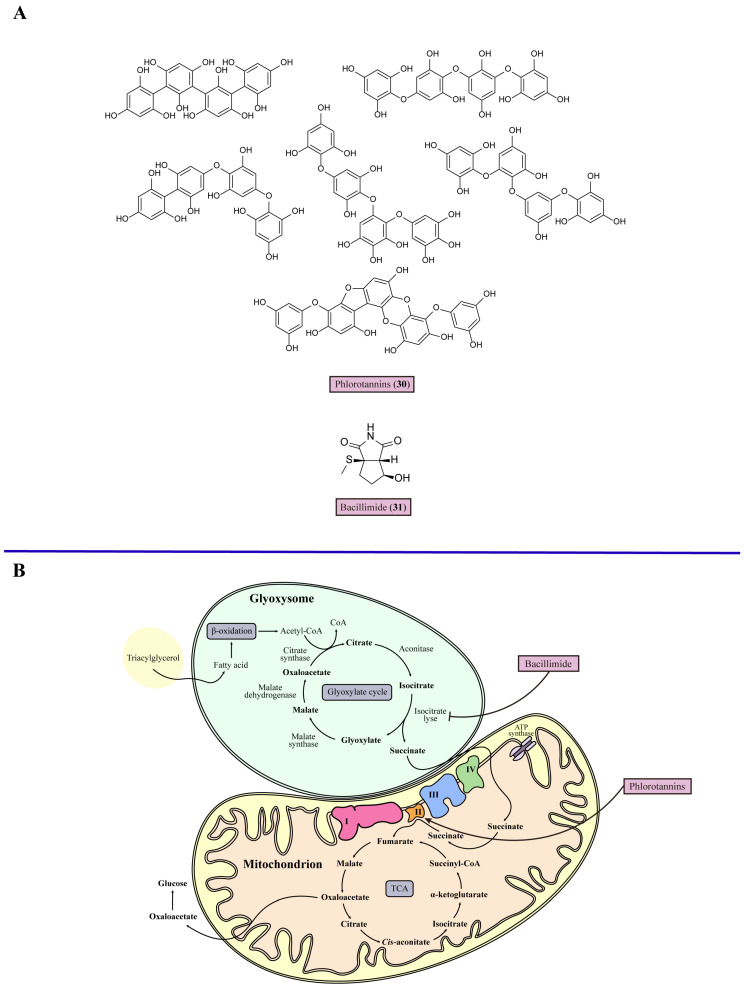
Chemical structures and detailed mechanisms of marine-derived metabolites targeting fungal mitochondria. (**A**) Chemical structures of phlorotannins (**30**) and bacillimide (**31**). (**B**) Fungal respiratory metabolism includes the glyoxylate cycle and TCA. Therein, the TCA is regulated by the respiratory chain consisting of Complexes I–IV. Phlorotannins stimulate Complex II to increase mitochondrial respiratory rate, leading to ROS accumulation and regulating mitochondrial membrane potential for mitochondrial dysfunction; bacillimide inhibits the transcription of isocitrate lyase to suppress the glyoxylate cycle.

For every kind of fungi, mitochondria are the hubs of respiration and metabolic activity, such as oxidative phosphorylation and the citric acid cycle, which are also involved in phospholipid metabolism [81]. Mitochondria impact multiple aspects of fungal pathogenesis, such as fungal growth, antifungal susceptibility, and the yeast-to-hypha transition [82]. Mitochondrial dysfunction decreases the activity of respiratory chain enzymes and the mitochondrial membrane potential, initially leading to damaged intracellular Ca^2+^ homeostasis and blocked β-oxidation of fatty acids. Then, cellular fatty acids accumulate, and oxidative stress increases, resulting in the oxidative damage of mitochondrial DNA. Thereby, mitochondrial biosynthesis is reduced, which further aggravates mitochondrial dysfunction, causing the death of fungal cells [83]. The respiratory chain is also called the electron transport chain, consisting of Complexes I–IV and a cyanide-insensitive alternative oxidase (AOX) in most fungal pathogens that are potential targets for mitochondrial dysfunction. In *C. albicans*, the respiratory chain inhibitor antimycin A (**27**) inhibits Complex III, which is one of the major sources of mitochondrial ROS accumulation, resulting in an increase in oxidative stress and causing the decreased proliferation of *C. albicans* [84]. For example, salicylhydroxamic acid (**28**) acts as an AOX inhibitor in *Trypanosoma brucei* (*T. brucei*) with moderate antifungal activity in vitro (EC_50_ = 15 μM) [85]. The glyoxylate cycle is important in pathogenic fungi for full virulence distributed in mitochondria, and is composed of several reactions in the tricarboxylic acid cycle (TCA) and two steps catalyzed by additional enzymes. There are three reactions in the TCA: the conversion of malate to oxaloacetate catalyzed by malate dehydrogenase, the transformation of oxaloacetate into citrate through citrate synthase, and the conversion of citrate to isocitrate by aconitase. Then, one of the additional enzymes, isocitrate lyase, cleaves isocitrate into glyoxylate and succinate. The other malate synthase converts glyoxylate and acetyl-CoA to malate 3 [86] (Figure 4). The enzyme isocitrate lyase, one of the principal enzymes in the glyoxylate cycle, is an important target. A mutant *C. albicans* strain lacking isocitrate lyase showed a remarkable decrease in virulence and persistence in systemic candidiasis mice [87]. Arylamidine T-2307 (**29**) selectively disrupts yeast mitochondrial function by inhibiting respiratory chain Complex III and Complex IV [88], suggesting that fungal mitochondria may serve as a viable target for antifungal agents.

Phlorotannins (**30**), extracted from brown seaweeds, displayed antifungal properties against common fungal pathogens, especially for *C. albicans* ATCC 10231, with MIC values of 15.6 mg/mL [89]. Further studies have showed that phlorotannins (**30**) stimulate the activity of mitochondrial succinate dehydrogenase, also known as electron transport chain Complex II, and increases the mitochondrial respiratory rate, which increases the generation of toxic intermediate species, including superoxide, hydrogen peroxide, and hydroxyl radicals called ROS (Figure 4). Some of these ROS escape from several enzymatic and non-enzymatic systems, contributing to free radical inactivation and leading to the accumulation of ROS, resulting in toxic effects that suppress fungal cell division. Furthermore, phlorotannins (**30**) have a dual mechanism for regulating the mitochondrial membrane potential. At higher phlorotannin (**30**) concentrations, the anti-apoptotic defenses in fungal cells were triggered, which increased the expression of anti-apoptotic proteins such as Bcl-2 and Bcl-xL, reducing oxidative stress and blocking cell death, and the expression of these anti-apoptotic proteins leads to quicker stabilization of the membrane potential taking place [90]. At lower phlorotannin (**30**) concentrations, anti-apoptotic defense mechanisms were not triggered, and instead, Ca^2+^ permeability took place, leading the fungal cell to recover from the hyperpolarized state [91]. Nevertheless, further studies are required on the effect of the dual mitochondrial membrane potential-regulating mechanism of phlorotannins (**30**) on antifungal activity. In conclusion, inducing mitochondrial dysfunction could be a feasible approach for targeted therapy for mycoses (Table 1). Bacillimide (**31**), one of the nitrogenous metabolites from the broth of marine actinomycete *Streptomyces bacillaris*, with a rare structural class cyclopenta[c]pyrrole-1,3-dione, displayed moderate inhibitory ability against *C. albicans* with with an MIC value 44.24 μM [92]. A growth assay revealed that 128 μg/mL bacillimide (**31**) almost completely inhibited the transcriptional level of isocitrate lyase in *C. albicans* to suppress the glyoxylate cycle (Figure 4, Table 1).

## 6. Inhibition of Vesicular Trafficking

**Figure 5:**
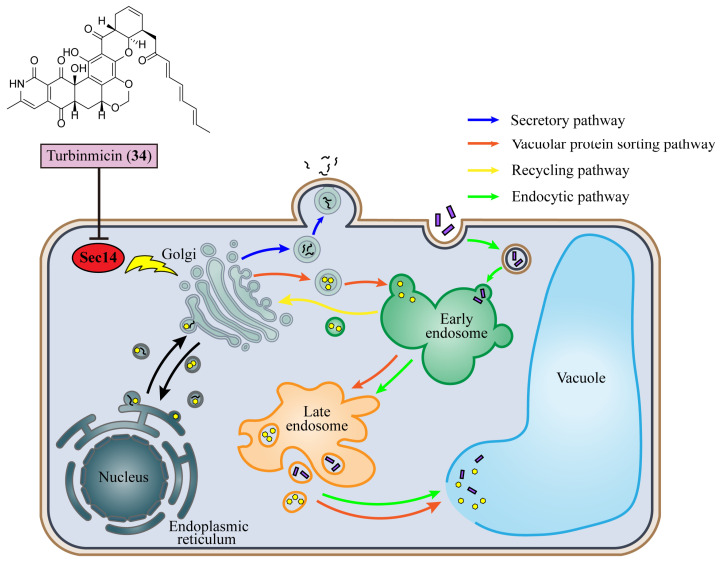
Representation of fungal intracellular vesicular trafficking and antifungal action of turbinmicin (**34**). Vesicular trafficking is a prominent pathway for conveying proteins into the target position. Proteins are synthesized at the endoplasmic reticulum and sorted based on their functions at the Golgi. Plasma membrane and extracellular proteins are transported into secretory vesicles (blue arrows); vacuolar proteins are conveyed through vacuolar protein sorting vesicles (orange arrows); the recycling pathway would transport partial proteins back to the Golgi to avoid degradation (yellow arrows); the endocytic pathway is used for internalized proteins being targeted into the vacuole (green arrows). Turbinmicin targets Sec14, a protein regulating the exportation of vesicles from the Golgi, to decrease the production of fungal extracellular vesicles for potent antifungal activity.

Vesicular trafficking is a prominent pathway in protein biosynthesis for the transport of proteins into their target positions. In fungal cells, extracellular, membrane, and organellar proteins are translocated to the endoplasmic reticulum during protein processing. Then, these proteins are directed to the Golgi apparatus for further modification. Proteins are sorted at the Golgi membrane, extracellular proteins are transported to the plasma membrane or external medium by the secretory pathway, and organellar proteins pass through endosomes for targeting the vacuole via the vacuolar protein sorting pathway. Conversely, membrane and extracellular proteins can be internalized by the endocytic pathway when proteins are transported to early endosomes, where they are sorted for those targeting vacuolar degradation and others following the recycling pathway directly to the Golgi to be remodified for the secretory pathway [93]. Sec14 is a phosphatidylinositol transfer protein regulating the exportation of vesicles from the Golgi in vesicular trafficking (Figure 5). Sec14 has a conserved structure with two lobes, comprising four antiparallel β-strands surrounded by two long α-helices. Meanwhile, the larger lobe possesses a phospholipid-binding pocket containing two octyl glucoside molecules [8,94]. In their 2019 study, Van Dijck et al. demonstrated that vesicular transport plays a role in the susceptibility of fungi to fluconazole (**32**). The combination of fluconazole (**32**) with the vesicular trafficking inhibitor sortins (**33**) resulted in the enhanced susceptibility of *Candida* species, indicating the potential utility of targeting vesicular transport in the treatment of fungal diseases [95].

An encouraging antifungal molecule has been discovered from the *Micromonospora* species of sea squirt, turbinmicin (**34**) [96]. In vitro, turbinmicin (**34**) had MIC values ranging from 0.03 to 0.5 μg/mL across most pathogenic fungi, including *C. auris*, *C. albicans*, *C. tropicalis*, *C. glabrata*, *A. fumigatus*, *Fusarium* species, and *Scedosporium* species. In invasive pulmonary aspergillosis mice, 1 mg/kg turbinmicin every 6 h of treatment reduced the *Aspergillus* fungal burden with a 1.5 log_10_ drop in a dose-dependent manner. 

Mode of action studies have shown that turbinmicin (**34**) impairs vesicle-mediated trafficking by targeting Sec14 (Figure 5). A docking study revealed that turbinmicin (**34**) binds the phospholipid binding pocket of Sec14. The heptacyclic ring system of turbinmicin (**34**) co-crystallizes with the picolinamide and octyl glucoside in a phospholipid binding pocket. Meanwhile, the polyene tail in the turbinmicin (**34**) structure is extended into the hydrophobic cleft vacant. Furthermore, turbinmicin (**34**) also exhibits activity against biofilms [97]. Biofilms are composed of protective extracellular polymeric substances, with key components of α-mannan, β-1,6-glucan, and β-1,3-glucan, conferring a critical adherence and drug resistance ability to fungal cells. Extracellular vesicles can deliver polymeric substances for biofilm construction [98,99]. After exposure to 2 μg/mL turbinmicin (**34**), *C. albicans* showed a more than 500% reduction in vesicle delivery. A nearly 300% reduction has also been observed in the mannan–glucan complex extracellular matrix at 40 μg/mL turbinmicin (**34**) treatment. Therefore, turbinmicin (**34**) could bind with Sec14 to interfere with the secretory pathway of vesicular trafficking in the Golgi and decrease the production of fungal extracellular vesicles, revealing a new approach to eradicating drug-resistant fungal pathogens (Table 1).

## 7. Inhibiting Efflux Pumps

**Figure 6:**
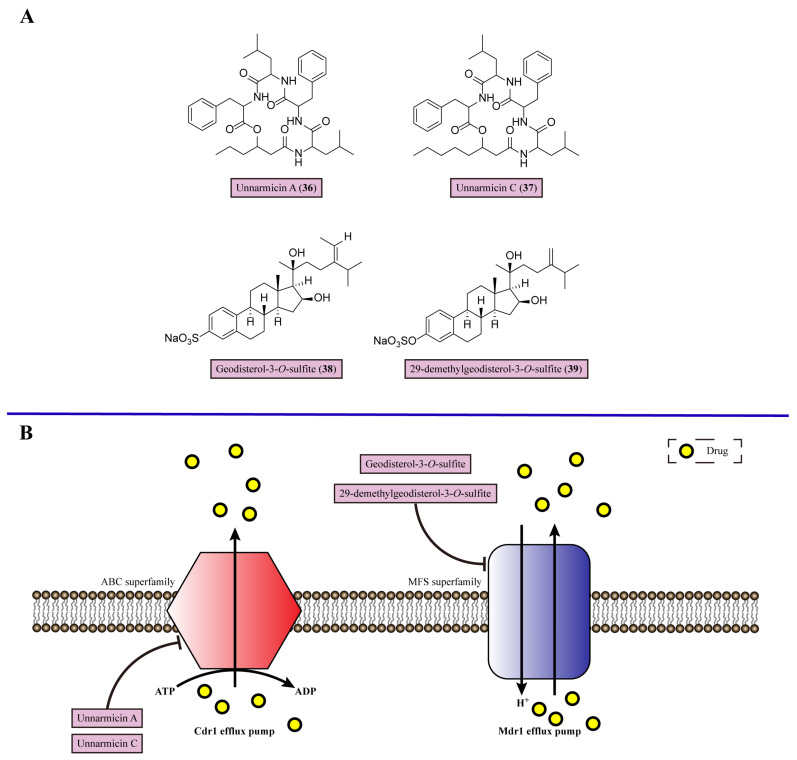
Chemical structures and detailed mechanisms of marine-derived metabolites targeting efflux pump. (**A**) Chemical structures of unnarmicin A (**36**), unnarmicin C (**37**), geodisterol-3-*O*-sulfite (**38**), and 29-demethylgeodisterol-*O*-sulfite (**39**). (**B**) Efflux pumps from the ABC and MFS superfamily mediate MDR in fungal infection. Unnarmicin A and unnarmicin C act as inhibitors against *C. albicans* ABC transporter Cdr1 efflux pump to attenuate azole resistance; geodisterol-3-*O*-sulfite and 29-demethylgeodisterol-*O*-sulfite target Mdr1 efflux pump in *C. albicans*, belonging to the MFS superfamily, to avoid fluconazole resistance.

Multidrug resistance (MDR) has been a major concern in fungal infection, which is mainly mediated by the ATP-binding cassette transporter (ABC) superfamily and major facilitator (MFS) superfamily. The overexpression of the ABC and MFS superfamilies would significantly strengthen drug efflux, leading to the decline in drug concentration in fungal cells (Figure 6). Therefore, it is unable to accumulate drugs intracellularly to reach toxic levels, resulting in drug-resistant properties [100]. Holzgrabe and colleagues conducted the synthesis of cerulenin derivatives to serve as inhibitors of efflux pumps, resulting in a reduction in MFS-mediated resistance to brefeldin A (**35**) by up to eight-fold in multidrug-resistant *C. albicans* [101].

Small molecule multidrug efflux pump inhibitors could block the activity of these drug pumps to accumulate the antifungal drug inside cells, performing suppression of fungal growth [102]. Unnarmicin A (**36**) and unnarmicin C (**37**) are derived from marine γ-proteobacterium, which specifically inhibit the ATPase activity of *C. albicans* ABC transporter Cdr1, an azole drug efflux pump in *C. albicans*, with MIC values of 0.495 and 0.688 μM, respectively [103] (Figure 6). Both unnarmicin A (**36**) and unnarmicin C (**37**) attenuate the azole resistance of azole-resistant *C. albicans* overexpressing *C. albicans* Cdr1, as the MIC of fluconazole (**32**) (80 μg/mL) is reduced to 10 μg/mL when 0.312 μM unnarmicin A (**36**) or 0.312 μM unnarmicin C (**37**) is present. Therefore, unnarmicin A (**36**) and unnarmicin C (**37**) are efflux pump inhibitors, which could be used as adjuvants for azole-resistant *C. albicans* therapy (Table 1).

Geodisterol-3-*O*-sulfite (**38**) and 29-demethylgeodisterol-3-*O*-sulfite (**39**) are isolated from marine sponge *Topsentia* species, targeting the overexpressed Mdr1 efflux pump in *C. albicans*, which belongs to the MFS superfamily [104] (Figure 6). These two new sulfated sterols reversed the efflux pump-mediated fluconazole (**32**) resistance, which showed significant synergy with fluconazole (**32**) and either of them against fluconazole-resistant *C. albicans* 1758 (FICI = 0.2 for both compounds) (Table 1).

## 8. Inhibiting Fungal Hyphal Growth

*C. albicans* is characterized by morphological plasticity, and can switch between the yeast, pseudohyphal, and hyphal growth forms, contributing to its virulence [105]. Of these, the filamentous hypha form is important for its infective stage, which could enhance its ability to invade host tissues and help fungi escape the phagocytosis of macrophages [106]. Moreover, the genes that control hyphal morphology are co-regulated with genes encoding virulence factors. As hypha-specific genes, hyphal wall protein 1 (*HWP1*) and Agglutinin-like protein 3 (*ALS3*) encode fungal cell wall proteins that are important for adhesion to host cells and iron acquisition [107,108]. So, suppressing the initiation of fungal hypha is an effective target for treating fungal infections [13].

**Figure 7:**
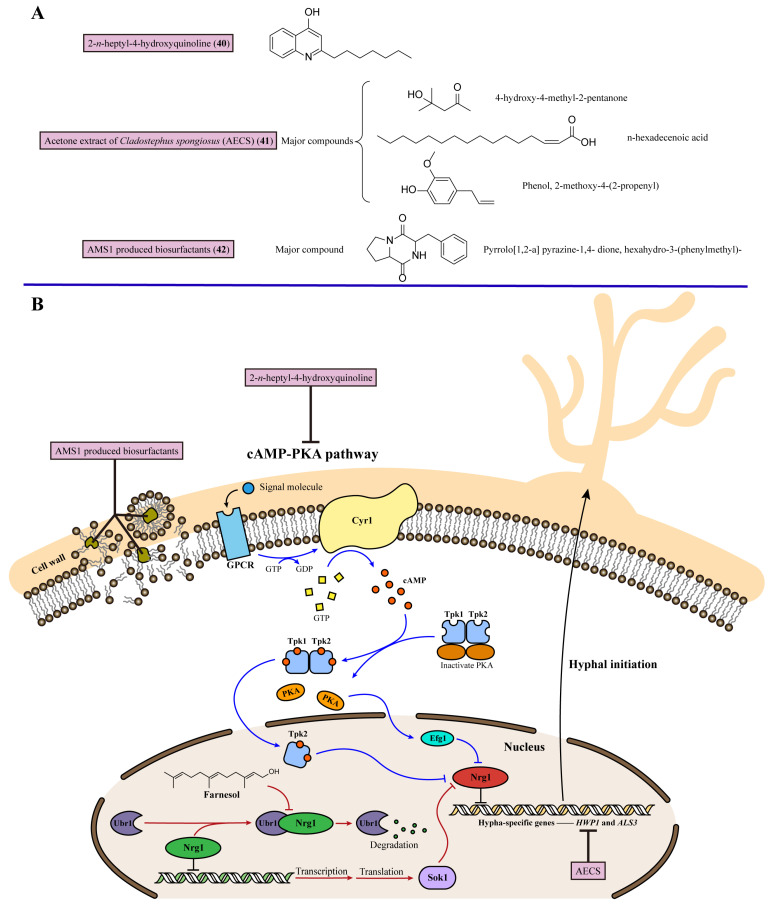
Chemical structures and detailed mechanisms of marine-derived metabolites targeting fungal hyphal growth. (**A**) Chemical structures of 2-*n*-heptyl-4-hydroxyquinoline- (**40**), AECS- (**41**), and AMS1-produced biosurfactants (**42**). (**B**) Hypha-specific gene transcriptional repressor Nrg1 controls the expression of *HWP1* and *ALS3*. Meanwhile, two pathways, cAMP-PKA (blue arrows) and Ubr1-mediated Cup9 degradation by farnesol (red arrows), are involved in the downregulation of Nrg1 during hyphal initiation. 2-*n*-heptyl-4-hydroxyquinoline decreases the expression of hypha-specific genes *HWP1* and *ALS3* by inhibiting cAMP-PKA pathway; AECS downregulates the expression of hypha-specific genes, including HWP1, ALS1, and SAP4, to suppress hyphal growth.

There are two key signaling pathways involved in *C. albicans* hyphal initiation by downregulating the hypha-specific gene transcriptional repressor Nrg1, which would lead to the yeast-to-hypha transition in *C. albicans*. One is the activation of the cAMP-PKA signaling pathway: the adenylyl cyclase Cyr1 stimulates cAMP production, which then activates protein kinase A (PKA) with two catalytic subunits, Tpk1 and Tpk2. Of these, Tpk2 could downregulate Nrg1. Moreover, the transcription factor Efg1 functions downstream of the cAMP-PKA pathway, and is required for the downregulation of *NRG1* expression. The other pathway is based on farnesol, which is a quorum-sensing molecule in *C. albicans*, inhibiting germ tube formation. When *C. albicans* is released from farnesol inhibition, the N-end rule E3 ligase Ubr1 degrades the Cup9 transcriptional repressor, then the rapid degradation of Cup9 transiently increases the expression of Sok1, whose major function is to downregulate Nrg1. Therefore, inhibiting the cAMP-PKA pathway and maintaining farnesol inhibition to upregulate Nrg1 transcription could block hyphal initiation in *C. albicans* [109] (Figure 7).

In 2019, 2-*n*-heptyl-4-hydroxyquinoline (**40**) was identified from marine *Streptomyces* species MBTG13 [110]. 2-*n*-heptyl-4-hydroxyquinoline (**40**) exhibited a potent inhibitory effect against the morphogenesis of *C. albicans* from yeast into hypha forms with IC_50_ values of 11.4 μg/mL. By semi-quantitative reverse transcription PCR analysis, 2-*n*-heptyl-4-hydroxyquinoline (**40**) regulated the cAMP-Efg1 pathway to control the hyphal growth of *C. albicans* (Figure 7). The cAMP-Efg1 pathway is one of the hypha-inducing signaling pathways, stimulated by Ras1 and transcription factor Efg1. This pathway mediates the expression of downstream hypha-specific genes in *C. albicans,* including *ALS3*, *ECE1*, and *HWP1*, and HWP1 is the most highly expressed gene encoded. *HWP1* and *ALS3* encode adhesins and are activated by the transcription regulator Efg1 during hypha formation. *C. albicans* cells incubated with 100 μg/mL 2-*n*-heptyl-4-hydroxyquinoline (**40**) showed dramatically reduced mRNA expression of *HWP1* and *ALS3* compared with untreated cells. In conclusion, 2-*n*-heptyl-4-hydroxyquinoline (**40**) inhibited the growth of *C. albicans* hypha through the cAMP-Efg1 pathway to decrease the expression of the hypha-specific genes *HWP1* and *ALS3* (Table 1). 

Promising anti-candidal activity of the acetone extract of *Cladostephus spongiosus* (AECS) (**41**) has been reported [111]. The treatment against *C. krusei* showed that AECS (**41**) inhibited the metabolism in matured *C. krusei* biofilms and prevented the formation of biofilms, as the values of the biofilm inhibitory concentration (BIC) of AECS (**41**) against *C. krusei* was 120 μg/mL. Moreover, AECS (**41**) caused cell deformation and the distortion of cell membranes, indicating its antibiofilm activity. AECS (**41**) also downregulated the expression of hyphal-specific genes, including *HWP1*, *ALS1*, and the fourth secreted aspartyl proteinase (*SAP4*), to suppress the hyphal growth of *C. krusei* (Figure 7). Furthermore, according to gas chromatography-mass spectrophotometer analysis, the major compounds in AECS (**41**), 4-hydroxy-4-methyl-2-pentanone, n-hexadecenoic acid, and phenol, 2-methoxy-4-(2-propenyl) could be active compounds for use in candidemia therapy, but further results from comprehensive activity studies are awaited (Figure 7, Table 1). 

Nithyanand (2021) et al. discovered a biosurfactant isolated from the marine bacterium AMS1, which displayed a disruptive effect against biofilms and inhibited the transition into hyphae [112]. A biosurfactant from AMS1 (**42**) inhibited the growth of *C. albicans* with an MIC value of 160 μg/mL. Moreover, the synergistic activity of biosurfactants with marine bacterial DNase and DNase I showed an obvious inhibition effect against the mature biofilms of *C. albicans*, about 85% and 79%, after 24 h of incubation. Moreover, pyrrolo[1,2-a] pyrazine-1,4-dione, hexahydro-3-(phenylmethyl)- is the major component in the AMS1-produced biosurfactant, which could be a potential candidate biosurfactant for antifungal agent development (Figure 7, Table 1).

## 9. Inhibiting Biofilm Formation

**Figure 8:**
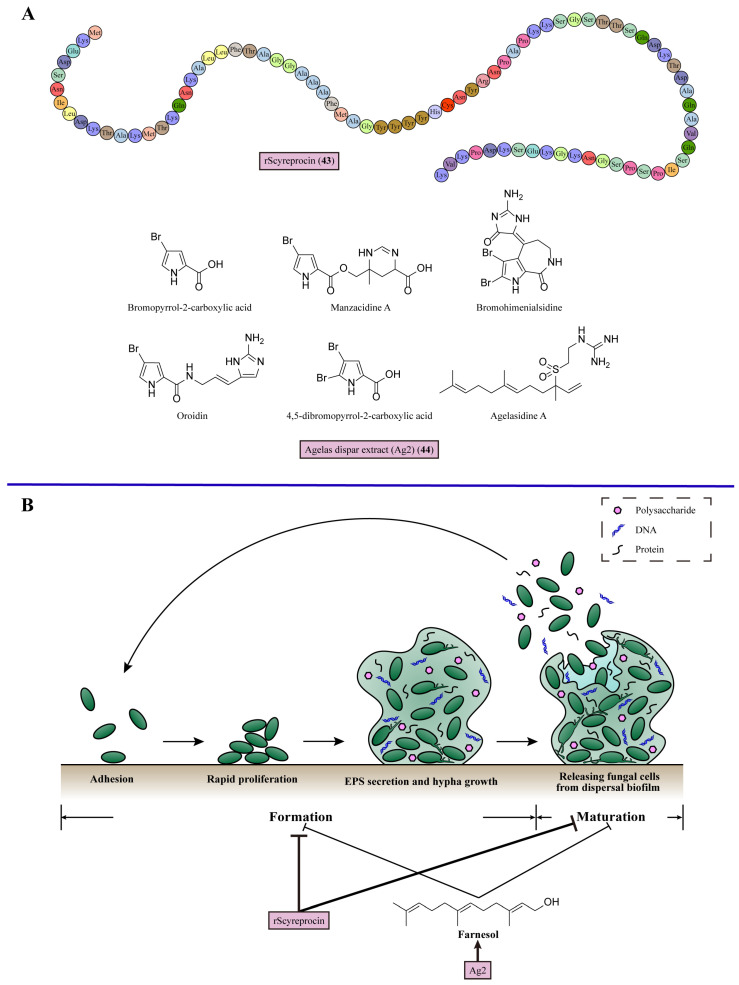
Chemical structures and detailed mechanisms of marine-derived metabolites targeting biofilm formation. (**A**) Chemical structures of rScyreprocin (**43**) and Ag2 (**44**). (**B**) The state of biofilm can be identified as formation or maturation. In the formation state, fungal cells attach to the surface and trigger rapid proliferation, and then cells are covered by secreted EPS, along with the growth of hypha. Fungal cells are released from dispersed biofilm for new biofilm formation during the maturation of biofilm. rScyreprocin suppresses the formation of biofilm and eradicates maturation of biofilm; Ag2 produces farnesol to prevent biofilm formation and maturation.

Biofilms occur when microbes adhere to abiotic or biotic surfaces to form communities encased in multiple extracellular polymeric substances (EPS) produced by these microbes. Compared with single microbial cells, microbial communities with biofilms have significantly increased resistance against antifungal treatment and the ability to escape from the host’s immune system defenses [113]. EPS are composed of the polymers of polysaccharides, proteins, and DNA. Abundant extracellular polysaccharides are major components, including the structures of α-mannan, β-1,6-glucan, and β-1,3-glucan [98]. There are four steps in biofilm formation. For *C. albicans*, cells first attach to the surface and then maintain rapid proliferation initially. Then, fungal cells secrete EPS until completely encased, along with the growth of hypha, meaning the maturation of biofilm. Finally, the dispersed biofilm releases *C. albicans* cells for further proliferation and formation of novel biofilms [114]. The factors known to disturb the formation of biofilms include the characteristics of adhering surfaces, nutrients, and quorum sensing. Quorum sensing is the foundation of biofilms, enabling communication between microbes through signaling molecules [115]. In the hyphal initiation mechanism, farnesol is one of the quorum-sensing molecules, which could inhibit the hyphal growth in *C. albicans* [109]. For biofilms, exogenous farnesol blocks the formation of the biofilm, affecting the adherence of the cells, the architecture of the biofilms, and the dispersal of the biofilms [115] (Figure 8). The combination of echinocandins with farnesol demonstrates increased inhibitory efficacy against *C. parapsilosis* biofilms, as evidenced by the significant reduction in the median MIC values of caspofungin and micafungin in combination with farnesol by 8–61- and 4–64-fold, respectively [116].

In 2020, Wang et al. isolated a peptide from the mud crab *Scylla paramamosain* and expressed it in *Escherichia coli* to obtain rScyreprocin (**43**), which displayed broad-spectrum antifungal and antibiofilm activities [117]. rScyreprocin (**43**) was susceptible to *C. neoformans* and *Candida* species (MIC = 1–32 μM). Meanwhile, a potent inhibitory effect against spore germination in *Aspergillus* species (MIC = 4–8 μM) was found with rScyreprocin (**43**) treatment. In the biofilm inhibition assay, 2–8 μM rScyreprocin (**43**) reduced the adhesion of *C. neoformans* in a concentration-dependent manner. rScyreprocin (**43**) also suppressed the formation of biofilms and eradicated mature biofilms in the treatment of *C. albicans* and *C. neoformans* (Figure 8, Table 1).

Resende-Stoianoff (2021) et al. discovered the antibiofilm activity of the extract of the sponge *Agelas dispar* (Ag2) (**44**) [118]. Ag2 (**44**) inhibited the growth of *Candida* strains with MIC values varying between 0.15625 and 2.5 mg/mL. In antibiofilm studies, Ag2 (**44**) at 2.5 mg/mL inhibited the formation of biofilms and disrupted mature biofilms of *C. krusei*, *C. glabrata*, and *C. parapsilosis*. A possible explanation for the antibiofilm activity of Ag2 (**44**) is a compound in the Ag2 fraction, Agelasidin A. This secondary metabolite of *Agelas dispar* could produce farnesol to prevent biofilm formation (Figure 8, Table 1).

**Table 1:** The characteristics of antifungal compounds and extracts derived from marine organisms.

No.	Metabolites	Mechanism	Source	Activity	Refs.
1	Q-Griffithsin (**9**)	Binds with α-mannan to break the outer layer of the fungal cell wall	Red alga *Griffithsia* species with glutamine substitution of Met78	*Candida albicans*, *Candida glabrata*, *Candida parapsilosis*, *Candida krusei*, and *Candida auris* (MIC = 6, 95, 24, 95, and 48 mg/mL)	[32]
2	15G256γ (**11**)	Inhibits the activity of chitin synthase to weaken fungal cell wall	Marine fungus *Hypoxylon oceanicum*	*Trichophyton rubrum*, *T. mentagrophytes*, *Epidermophyton floccosum*, *Microsporum audoinii*, *C. albicans*, *C. parapsiliosis*, and *C. glabrata* (MIC = 2–16 μg/mL)	[44]
3	Tubingenoic anhydride A (**12**)	Suppresses the expression of *mas-1* mediating part of chitin synthase expression	Fungus *Aspergillus tubingensis* OY907 from sponge *Ircinia variabilis*	*Neurospora crassa* (MIC = 330 μM)	[45]
4	Puupehenone (**15**)	Acts as an Hsp90 inhibitor to block cell wall integrity pathway	Marine sponge *Hyrtios* species	*Cryptococcus neoformans*, *C. glabrata*, and *C. albicans* (puupehenone + caspofungin FICI = 0.38, 0.48, and 0.39)	[51]
5	Plakortide F acid (PFA) (**17**)	Disrupts fungal intracellular calcium ion homeostasis	Marine sponge *Plakortis halichondrioides*	*C. albicans*, *C. neoformans*, and *Aspergillus fumigatus* (MIC = 0.08, 2.5, and 5.00 μg/mL)	[57]
6	Theonellamide G (**18**)	Binds with the 3β-OH group in ergosterol	Marine sponge *Theonella* species	Wide-type *C. albicans* and amphotericin B-resistant *C. albicans* (IC_50_ = 4.49 and 2.0 μM)	[63]
7	3-(3-((12-azidododecyl)oxy)propyl)-1-benzylpyridin-1-ium chloride (**19**)	Binds with membrane ergosterol	Marine sponges of the Haplosclerida order	*C. albicans*, *C. glabrata*, *C. krusei*, and *Candida tropicalis* (MIC = 3.9–7.8 μg/mL)	[64]
8	Amantelide A (**20**)	Recognizes ergosterol to bind with membrane	Marine gray cyanobacteria Oscilliatoriales	*Saccharmomyces cerevisiae* and *Schizosaccharomyces pombe* (MIC = 50 and 12.5 μM)	[65]
9	Neothyonidioside (**21**)	Forms a large complex with ergosterol to reduce the ability of the fungal membrane to bend and form multivesicular body vesicles	Sea cucumber *Australostichopus mollis*	*S. cerevisiae* (MIC = 1 μM)	[66]
10	Amphidinol 3 (AM3) (**23**)	Recognizes 3β-OH group in ergosterol through hydrogen bonding to permeabilize the fungal cell membrane	Dinoflagellate *Amphidinium klebsii*	*Aspergillus Niger* (MEC = 9.0 μg/disk)	[68]
11	Oceanapiside (**25**)	Blocks sphingolipid biosynthesis in fungi	Marine sponge *Oceanapia phillipensis*	*C. glabrata* (MIC = 10 μg/mL)	[75]
12	MMGP1 (**26**)	Internalized into the cytosol to form MMPG1–DNA complex, interfering with transcription	Marine metagenome	*C. albicans* (MIC = 0.57 μM)	[80]
13	Phlorotannins (**30**)	Stimulates the activity of electron transport chain Complex II and regulates mitochondrial membrane potential to induce mitochondrial dysfunction	Brown seaweeds *Cytoseira nodicaulis*, *Cystoseira usneoides*, and *Fucus spiralis*	*C. albicans* (MIC = 15.6 mg/mL)	[89]
14	Bacillimide (**31**)	Inhibits the transcription of isocitrate lyase to suppress the glyoxylate cycle	Marine actinomycete *Streptomyces bacillaris*	*Candida albicans* (IC_50_ = 44.24 μM)	[92]
15	Turbinmicin (**34**)	Binds with Sec14 to interfere with the secretion during vesicular trafficking in the Golgi and decreases the production of fungal extracellular vesicles	Sea squirt *Micromonospora* species	*C. auris*, *C. albicans*, *C. tropicalis*, *C. glabrata*, *A. fumigatus*, *Fusarium* species, and *Scedosporium* species (MIC = 0.03–0.5 μg/mL)	[96]
16	Unnarmicin A (**36**)	Targets Cdr1 efflux pump in *C. albicans*	Marine γ-proteobacterium	Azole-resistant *C. albicans* (unnarmicin A + fluconazole MIC = 10 μg/mL)	[103]
17	Unnarmicin C (**37**)	Targets Cdr1 efflux pump in *C. albicans*	Marine γ-proteobacterium	Azole-resistant *C. albicans* (unnarmicin C + fluconazole MIC = 10 μg/mL)	[103]
18	Geodisterol-3-*O*-sulfite (**38**)	Targets Mdr1 efflux pump in *C. albicans*	Marine sponge *Topsentia* species	Fluconazole-resistant *C. albicans* (Geodisterol-3-*O*-sulfite + fluconazole FICI = 0.2)	[104]
19	29-demethylgeodisterol-3-*O*-sulfite (**39**)	Targets Mdr1 efflux pump in *C. albicans*	Marine sponge *Topsentia* species	Fluconazole-resistant *C. albicans* (29-demethylgeodisterol-3-*O*-sulfite + fluconazole FICI = 0.2)	[104]
20	2-*n*-heptyl-4-hydroxyquinoline (**40**)	Regulates cAMP-Efg1 pathway to decrease expression of the hypha-specific genes *HWP1* and *ALS3*	Marine *Streptomyces* species MBTG13	Hyphal form of *C. albicans* (IC_50_ = 11.4 μg/mL)	[110]
21	Acetone extract of *Cladostephus spongiosus* (AECS) (**41**)	Downregulates the expression of the hypha-specific genes *HWP1*, *ALS1*, and *SAP4*	Marine mactoalgal *Cladostephus spongiosus*	*C. krusei*, *C. glabrata*, *C. parapsilosis*, and *C.albicans* (MIC = 80, 90, 100, and 90 μg/mL)	[111]
22	AMS1 produced biosurfactants (**42**)	Acts as a biosurfactant to inhibit the transition from yeast to hyphae	Marine bacterium AMS1	*C. albicans* (MIC = 160 μg/mL)	[112]
23	rScyreprocin (**43**)	Suppresses biofilm formation and eradicates mature biofilms	Mud crab *Scylla paramamosain*	*C. neoformans*, *C. albicans*, *C. krusei*, *C. parapsilosis*, *C. tropicalis*, *N. crassa*, *Fusarium* species and *Aspergillus* species (MIC = 1–32 μM)	[117]
24	*Agelas dispar* extract (Ag2) (**44**)	Produces farnesol to prevent biofilm formation	Marine sponge *Agelas dispar*	*C. albicans*, *C. tropicalis*, *C. krusei*, *C. glabrata*, and *C. parapsilosis* (MIC = 0.15625–2.5 mg/mL)	[118]

## 10. Discussion

Marine-derived metabolites exhibit a high level of diversity and abundance. These compounds demonstrate a range of activities, such as antifungal properties [119,120,121]. The potential for discovering novel antifungal agents with potent activity, reduced toxicity, and decreased resistance from marine sources is promising. For example, the metabolite puupehenone (**15**) from marine sponges has shown efficacy in overcoming cytotoxicity in caspofungin-resistant strains of *C. neoformans*, *C. glabrata*, and *C. albicans*, indicating the potential for addressing the growing issue of fungal resistance using marine-derived compounds [51]. A novel antifungal mechanism has been discovered in the sea squirt-derived compound turbinmicin (**34**) [97]. By targeting vesicular trafficking in fungal cells, turbinmicin (**34**) exhibits potent broad-spectrum antifungal properties, presenting a promising alternative mechanism for the development of novel antifungal agents.

## 11. Conclusions

Discovering novel antifungals from marine organisms is a feasible method. This rich source of secondary metabolites provides different antifungal activities and mechanisms. These mechanisms involve targeting a range of cellular components, including the cell wall, cell membrane, mitochondria, chromosomes, and drug efflux pumps, as well as various biological processes such as vesicular trafficking and the inhibition of hyphal and biofilm growth. These potential active compounds require further in vivo experiments to verify their antifungal abilities. In summary, the utilization of secondary metabolites from marine organisms shows promise in the treatment of IFDs.
